# The long non-coding RNA TP73-AS1 modulates HCC cell proliferation through miR-200a-dependent HMGB1/RAGE regulation

**DOI:** 10.1186/s13046-017-0519-z

**Published:** 2017-04-12

**Authors:** Shaling Li, Yan Huang, Yun Huang, Yongming Fu, Daolin Tang, Rui Kang, Rongrong Zhou, Xue-gong Fan

**Affiliations:** 1 Hunan Key Laboratory of Viral Hepatitis, Department of Infectious Disease, Xiangya Hospital, Central South University, Changsha, 410008 China; 2 Department of Surgery, Xiangya Hospital, Central South University, Changsha, 410008 China; 3 Department of Surgery, University of Pittsburgh, Pittsburgh, 15260 USA

**Keywords:** lncRNA, TP73-AS1, miR-200a, HCC, HMGB1, Proliferation

## Abstract

**Background:**

P73 antisense RNA 1 T (non-protein coding), also known as TP73-AS1, is a long non-coding RNA (lncRNA) which is involved in cell proliferation and the development of tumors. However, the exact effects and molecular mechanisms of TP73-AS1 in hepatocellular carcinoma (HCC) progression are still unknown. The present study is aimed to investigate the detailed functions and the mechanism of TP73-AS1 in regulation of HCC cell proliferation.

**Methods:**

TP73-AS1 expression in HCC tissues and cell lines was determined using real-time PCR assays; the correlation of TP73-AS1 expression with clinicopathological features of HCC was analyzed. The functions of TP73-AS1 in regulation of HCC cell proliferation was evaluated using MTT and BrdU assays. The candidate upstream miRNAs of HMGB1 were screened using miRcode, miRWalk, miRanda and Target scan, verified using real-time PCR assays. The interaction between TP73-AS1 and miR-200a was confirmed using Luciferase report gene assays. The proten levels of HMGB1 signaling-related factors in response to co-processing TP73-AS1 knockdown and miR-200a inhibition were determined using Western blot assays and ELISA. Further, miR-200a, HMGB1 mRNA and RAGE mRNA and their correlations in HCC tissues were determined.

**Results:**

TP73-AS1 was upregulated in HCC tissues and cell lines. High TP73-AS1 expression was correlated with worse clinicopathological features, poorer prognosis and shorter survival. Knockdown of TP73-AS1 inhibited the HCC proliferation and the expression levels of HMGB1, RAGE and NF-κB in HCC cells. By using online tools, we screened out several candidate upstream miRNAs of HMGB1, among which miR-200a overexpression inhibited HMGB1 mRNA expression the most significantly. By using luciferase assays, we confirmed that miR-200a could directly bind to TP73-AS1 and the 3’UTR of HMGB1; TP73-AS1 competed with HMGB1 for miR-200a binding. MiR-200a inhibition could up-regulate HMGB1, RAGE, NF-κB expression as well as NF-κB regulated cytokines levels, which could be partially restored by si-TP73-AS1. In HCC tissues, miR-200a was down-regulated while HMGB1 and RAGE were up-regulated; TP73-AS1 was inversely correlated with miR-200a, while positively correlated with HMGB1 and RAGE, respectively.

**Conclusion:**

Our data indicated that TP73-AS1 might be an oncogenic lncRNA that promoted proliferation of HCC and could be regarded as a therapeutic target in human HCC.

**Electronic supplementary material:**

The online version of this article (doi:10.1186/s13046-017-0519-z) contains supplementary material, which is available to authorized users.

## Background

Liver cancer is the sixth most common cancer in the world, with 782,000 new cases diagnosed in 2012 alone. Hepatocellular carcinoma (HCC) is the dominant histological type of liver cancer, and accounts for about 80% of primary liver cancer occurrences [1]. In China specifically, HCC is a common type of cancer, and is the second highest cause of mortality in the country. More than 110,000 people die from liver cancer each year, accounting for 45% of all HCC deaths worldwide [2–4]. Despite therapeutic advances, the five-year survival rate of HCC is still below 5% [5–8]. HCC is the result of a multistep process which involves the accumulation of several structural and genomic alterations, gene expression profile and intracellular signal pathway alterations [9, 10]. Hence, it is critical to clearly understand these alterations and develop novel strategies for the early diagnosis, prognosis prediction and therapeutic target of patients with HCC.

Human genome sequence data indicates that more than 90% of the DNA sequences actively transcribed but only 2% of it encodes proteins, thus the majority of transcripts are referred to as non-coding RNAs (ncRNAs) [11, 12]. Small non-coding RNAs such as microRNAs have been studied extensively and their roles in gene regulation and cell function have been elucidated innumerous cancers [12]. Recent studies have shown that lncRNAs play important roles in both normal development and diseases including cancer [13]. LncRNAs have emerged as new players in cancer research and several studies have shown that some lncRNAs function as oncogenes, tumor suppressor genes or both, depending on the circumstance [14].

Several lncRNAs have been reported to be involved in cancers. Urothelial cancer-associated 1 (UCA1) lncRNA has been revealed significantly elevated in tongue squamous cell carcinoma tissues (*P* < .0001) and was statistically correlated with lymph node metastasis (*P* = .0371). Over-expression of UCA1 lncRNA could promote metastatic but not proliferation ability of TSCC cells [15]. LncRNA HULC enhances epithelial-mesenchymal transition to promote tumorigenesis and metastasis of HCC via the miR-200a-3p/ZEB1 signaling pathway [16]. LncRNA BANCR expression was remarkably increased in HCC tissues, BANCR downregulation in Hep3B cells impaired cell proliferation, promoted cell apoptosis, reduced cell invasion and migration, led to downregulated vimentin, and upregulated E-cadherin protein levels [17].

TP73-AS1, a lncRNA transcribed from chromosome 1p36, has been reported to be associated with cell proliferation and tumor progress [18]. Previous study predicted that TP73-AS1 might be up-regulated in HCC cells through Bioinformatics database [19]. Although emerging evidence has suggested that TP73-AS1 associated with cancer progression and prognosis, the detailed role of TP73-AS1 in HCC and the underlying mechanism still remains unclear.

LncRNAs exert their functions through different mechanisms, a major one of them is the interaction with miRNAs [20]. In addition, miRNAs themselves have been regarded as essential regulators in pathology of cancers [21, 22]. The miR-200 family consisting of 5 members (miR-200a, −200b, −200c, −141, −429) is an emerging miRNA family that has been shown to play crucial roles in cancer initiation and metastasis, and potentially be important for the diagnosis and treatment of cancer [23]. It was found that forced expression of miR-200a in meningioma cells reduced xenograft tumor growth when injected into the flanks of SCID [24]. Additionally, miR-200a has also been shown to directly target the pro-angiogenic ligands IL-8 and chemokine (C-X-C motif) ligand 1 (CXCL1) to regulate angiogenesis in ovarian cancer [25]. Whether miR-200a is involved in HCC? Can TP73-AS1 interact with miR-200a to modulate HCC progression? These remain to be investigated.

In this study, we report an interaction between TP73-AS1 and miR-200a which regulates HCC cell growth through directly targeting HMGB1. Our findings provide a novel understanding of the role of TP73-AS1 and miR-200a in HCC cell proliferation and the mechanism involved.

## Methods

### Cell lines

The human HCC cells, HCCLM3, MHCC97L, SMMC7722, Hep3B and HepG2, and normal hepatocyte THLE-3 were purchased from American Type Culture Collection (ATCC).

### Tissue specimens

Eighty-four paired HCC specimens and corresponding adjacent non-tumor tissues were collected from tumor surgical resection in the Xiangya Hospital (the Central South University, Changsha, China). And all the human tissues were obtained with informed consent and this study was approved by the Medical Ethics Committee of Xiangya hospital at Central South University.

### Cell transfection

All the miRNA mimics, inhibitor and siRNAs were synthesized by Genepharma Company (Shanghai, China). Oligonucleotide and plasmid transfection were conducted by using the Lipofectamine™ 2000 transfection reagent (Invitrogen, USA), followed by the protocol recommended by the manufacturer. After 48 h transfection, the cells were collected and used for further investigations.

### MTT assay

Following transfection, cell proliferation was assessed using 3-(4,5-dimethyl-2-thiazolyl)-2,5-diphenyl-2-H-tetrazolium bromide (MTT) assay (Promega Corporation, Madison, WI, USA), according to the manufacturer’s instructions. Cells were seeded into 96-well plates at a density of 5000 cells per well. After 24 h, cells were transfected with si-NC or si-TP73-AS1. After an additional 24 h, 20 μl of 5 mg/mL MTT was added into each well and incubated for 4 h in a humidified incubator. The supernatant was then discarded, and 200 μL of DMSO was added to each well to dissolve the formazan. The optical density (OD) was measured at 490 nm. The viability of the cells without any treatment was defined as 100%, and the viability of other groups was calculated separately from that of the sham cells.

### BrdU cell proliferation assay

By measuring 5-Bromo-2-deoxyUridine (BrdU) incorporation, the DNA synthesis in proliferating cells was determined. BrdU assays were conducted at 48 h after HCC cells were transfected with si-NC or si-TP73-AS1. Cells were seeded in 96-well culture plates at a density of 2 × 10^3^ cells/well, cultured for 48 h, then incubated with a final concentration of 10 μM BrdU (BD Pharmingen, San Diego, CA, USA) for 2 h. When the incubation period ended, the medium was removed, the cells were fixed for 30 min at RT, incubated with peroxidase-coupled anti-BrdU-antibody (Sigma-Aldrich) for 60 min at RT, washed three times with PBS, incubated with peroxidase substrate (tetramethylbenzidine) for 30 min, and the 450 nm absorbance values were measured for each well. Background BrdU immunofluorescence was determined in cells not exposed to BrdU but stained with the BrdU antibody.

### Cell clone formation assay

HCC cells were seeded and transfected with si-NC or si-TP73-AS1. Cells were suspended in RPMI-1640 containing 0.35% low-melting agarose and plated onto 0.6% agarose in six-well culture plates at a density of 1 × 10^5^ cells per dish. The plates were incubated for two weeks at 37 °C in a 5% CO_2_ incubator, and the number of colonies was counted microscopically after staining with 0.1% crystal violet solution. Colonies with more than 50 cells were manually counted.

### RNA extraction and real-time PCR

Total RNA was isolated by TRIZOL Reagent (Invitrogen, USA) following the manufacturer’s instructions. After RNA extraction, RNA samples were reversely transcribed by High Capacity cDNA Reverse Transcription Kit (Applied Biosystems, USA). The Fast Start Universal SYBR Green Master (Roche, USA) was applied for the quantitative RT-PCR. The relative fold changes of candidate genes were analyzed by using 2^−ΔΔ^CT method. Primers were shown in Additional file 1: Table S1.

### Western blot

Cell lysates were lysed by RIPA buffer (Sigma-Aldrich, USA) with Complete Protease Inhibitor Cocktail (Roche, USA). Cell lysates were transferred to 1.5 mL tube and kept at −20 °C before use. SDS-PAGE was conducted to separate the cellular proteins. And all the cellular proteins within this study were separated by 5% stacking gel and 10% running gel. The molecular weight of candidate proteins was referred to the information of the Pre-stained SeeBlue rainbow marker (Invitrogen, USA) loaded in parallel. The membranes were probed with the following antibodies: HMGB1 (Abcam, MA, USA), RAGE (Abcam, MA, USA), NF-κB (Abcam, MA, USA) and β-actin (Sigma, USA). The blots were detected on Kodak film developer (Fujifilm, Japan). The gradation analysis of the films was performed using Image J software (National Institutes of Health, Bethesda, MD, USA).

### ELISA

Cells (1 × 10^4^ cells per well) cultured in 96-well plates and transfected with miR-200a inhibitor or si-TP73-AS1 for 48 h. Then the culture media from each group was collected and stored at −80 °C until use. The levels of TNF-αNF-e levels of r in the media were measured using the Human TNF-α, IL-6, IL-1β/IL-1 F2 Quantikine ELISA kits, respectively (R&D systems, lnc., USA) according to the manufacturer’s instructions.

### Luciferase reporter assay

By using of the Lipofectamine™ 2000 transfection reagent, HepG2 cells cultured in 24 well plates were co-transfected with luciferase reporter plasmids (wt-HMGB1, mut-HMGB1 containing miR-200a binding site, wt-TP73-AS1, mut-TP73-AS1 containing miR-200a binding site1 or site2) and miRNA mimics or inhibitor as well as the internal control pRSV-β-Galactosidase vector. After 48 h post-transfection, HepG2 cells were lysed by lysis buffer (25 mMTris-phosphate, 1% Triton X-100, 1 mM DTT, 2 mM EDTA, 10% Glycerol, pH = 27.8). After centrifugation at 14,000 rpm for 3 min, the supernatant was transferred to a new 1.5 mL tube. The luciferase activity was monitored by mixing 50 μL supernatant with 50 μl luciferase assay buffer (265 μM ATP, 2.70 mM MgSO4, 1.07 mM MgCl2, 135 μM Coenzyme A, 20 mM Tricine, 0.1 mM EDTA, 33.3 mM DTT, 235 μM D-Luciferin) by using the Gloxmax 20/20 Luminometer (Promega). The β-Galactosidase activity from the pRSV-β-Galactosidase vector was used for the normalization of the luminescence levels. O-nitrophenyl- β-galactoside (ONPG) colorimetric assays were performed to measure the β-Galactosidase activity. The β-Galactosidase activity was evaluated by the measurement of o-nitrophenol by using the ELISA plate reader (Bio-Rad, USA) at the wavelength of 490 nm.

### Statistics

Experiments were done in triplicate. Experimental results are presented as Mean ± SD. Comparisons between two groups were conducted using two-tail Student’s *T*-test and differences were considered to be statistically significant when *P* value is less than 0.05.

## Results

### High lncRNA-TP73-AS1 expression in HCC was correlated with poorer prognosis

**Fig. 1:**
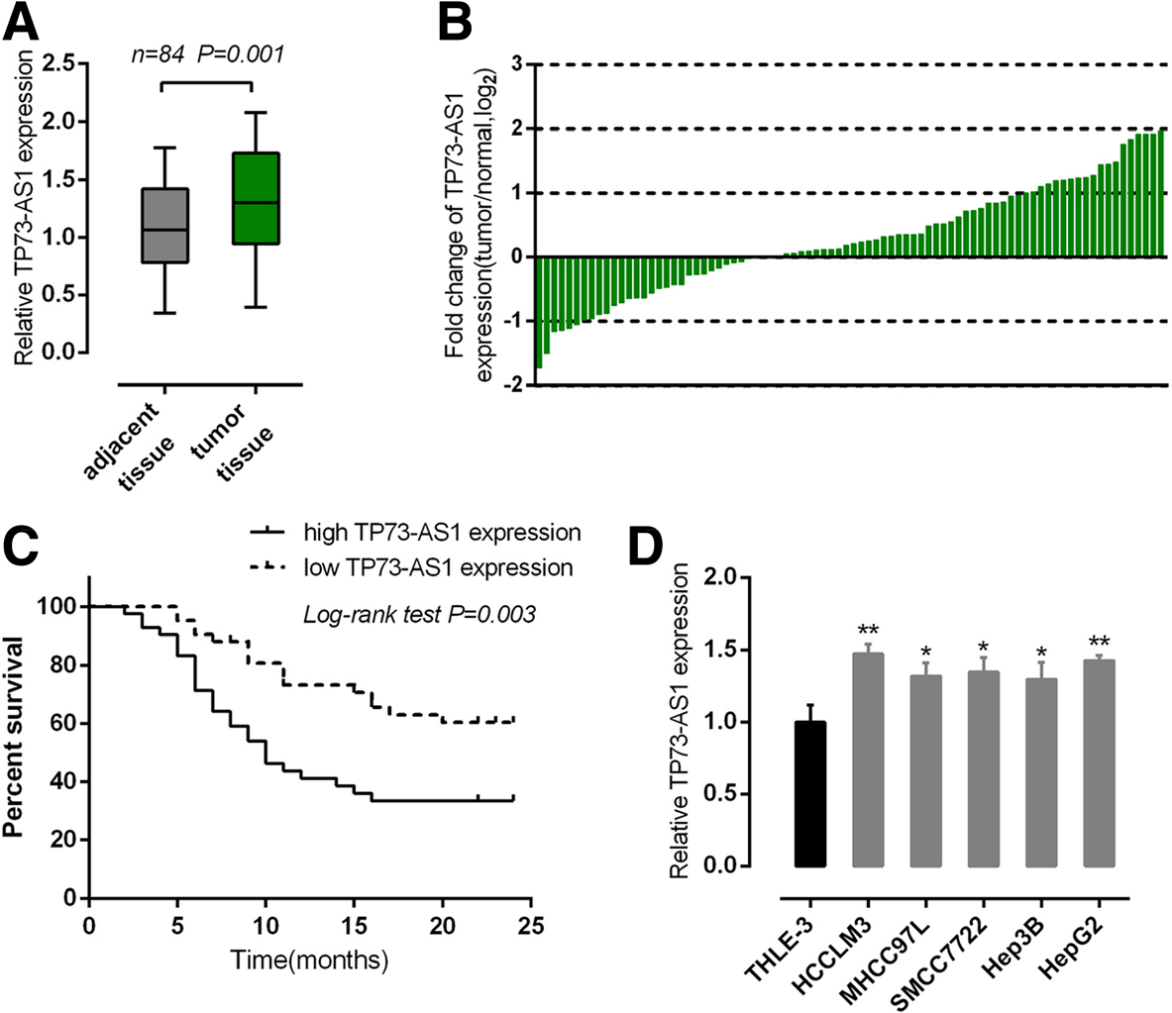
High lncRNA-TP73-AS1 expression in HCC was correlated with poorer prognosis **a** TP73-AS1 expression in a large panel of 84 paired cases of HCC tissues and adjacent normal tissues was determined by using real-time PCR. **b** Expression of TP73-AS1 in 84 pairs of HCC tissues and their corresponding adjacent non-tumorous tissues (ANTs) in a training cohort. Expression level of TP73-AS1 was determined by real-time PCR and normalized to U6. Fold change was analyzed using the formula 2-(ΔΔCT [HCC/ ANT]). **c** Kaplan-Meier overall survival curves for 84 patients with HCC classified according to relative TP73-AS1 expression level. **d** TP73-AS1 expression in five human HCC cell lines, HCCLM3, MHCC97L, SMMC7722, Hep3B and HepG2 was upregulated compared to normal cell line, THLE-3. *P < 0.05, **P < 0.01

Initially the expression levels of TP73-AS1 in 84 paired samples (HCC specimens and corresponding adjacent non-tumor tissues) were examined using real-time PCR. Results showed that TP73-AS1 expression was significantly higher in tumor tissues compared to mixed normal tissues (Fig. 1a). To validate this result, we performed quantitative real-time PCR in 84 cases of HCC tissues and adjacent normal tissues in training cohort. Compared to the corresponding normal tissues, TP73-AS1 showed to be significantly up-regulated (more than 2-fold [i.e., log_2_ (fold change) > 2]) in 51 HCC cases (>60.71%). 84 cases of HCC tissues were divided into two groups: a high TP73-AS1 expression group (above the median TP73-AS1 expression, *n* = 42) and a low TP73-AS1 expression group (below the median TP73-AS1 expression, *n* = 42). High expression of TP73-AS1 in HCC showed to be related with larger tumor size (*P* = 0.028), bigger tumor nodule number (*P* = 0.049) and advanced TNM stage (*P* = 0.002) as exhibited in Table 1. To determine the potential relationship between TP73-AS1 expression and the patients’ prognosis, Kaplan-Meier analysis and log-rank test were used to evaluate the effects of TP73-AS1 expression on overall survival (OS). The results indicated that patients with higher TP73-AS1 expression had a significantly poorer prognosis compared to patients with lower TP73-AS1 expression (*P* = 0.003) (Fig. 1c). A COX risk proportional regression model was further used to analyze the survival and pathological characteristics of 84 patients. The results of univariate analysis showed that tumor nodule number, TNM stage, BCLC staging and TP73-AS1 expression caused significant difference in survival time; the results of multivariate analysis showed that TNM stage (HR = 0.516; 95% CI: 0.261–0.902) and TP73-AS1 expression (HR = 2.249; 95% CI: 1.141–4.430) caused differences in survival time were statistically significant (Table 2). Then TP73-AS1 expressions in five human HCC cell lines, HCCLM3, MHCC97L, SMMC7722, Hep3B, HepG2 and a normal hepatocyte THLE-3 as control were determined using real-time PCR. Results showed that in all human HCC cell lines, expression levels of TP73-AS1 were upregulated compared to control group, among which TP73-AS1 were more significantly upregulated in HCCLM3 and HepG2 cells (Fig. 1d). These two cell lines were chosen for further experiments. The above data suggested that TP73-AS1 expressed at a higher level in HCC tissues and cell lines, and its high expression is related to larger tumor size, bigger tumor nodule number, advanced TNM stage and a shorter overall survival. High TP73-AS1 expression in HCC is related with poorer prognosis; however, the detailed role of TP73-AS1 in HCC progression still remains unclear.

**Table 1:** The Correlations of TP73-AS1 with Clinicopathological Features of HCC

Clinicopathologic Variable		TP73-AS1		*p*-value
		high expression	low expression	
Age	<50	23	22	0.827
	≥50	19	20	
Gender	Female	12	14	0.637
	Male	30	28	
Tumor size (cm)	<5	15	25	0.028
	≥5	27	17	
Tumor nodule number	Multiple (≥2)	26	17	0.049
	Solitary	16	25	
Venous invasion	Absence	19	18	0.826
	Presence	23	24	
TNM	I + II	12	21	0.002
	III + IV	30	26	
BCLC staging	0-A	8	16	0.132
	B	10	13	
	C	13	11	
	D	11	4	
Liver function	Child-Pugh A	17	24	0.126
	Child-Pugh B	25	18

**Table 2:** Univariate and Multivariate Analysis of Factors Associated with Overall Survival

Variable	n	Univariate Analysis		Multivariate Analysis	
		P	HR (95% CI)	P	HR (95% CI)
Age (year)		0.162		N.A	
<50	45		1.011 (1.000–1.034)		
≥50	39		1		
Gender		0.856		N.A	
Female	26		1		
Male	58		1.062 (0.561–2.013)		
Tumor size (cm)		0.39		N.A	
<5	40		0.771 (0.424–1.397)		
≥5	44		1		
Tumor nodule number		0.04		0.664	
Multiple (≥2)	43		1.910 (1.033–3.564)		1.166 (0.583–2.333)
Solitary	41		1		1
Venous invasion		0.522		N.A	
Absence	37		1		
Presence	47		1.217 (0.671–2.224)		
TNM		0.038		0.045	
I + II	33		0.508 (0.217–0.963)		0.516 (0.261–0.902)
III + IV	56		1		1
BCLC staging		0.029		0.129	
0-A	24		0.322 (0.122–0.891)		0.442 (0.154–1.267)
B	23				
C	24				
D	15		1		1
Liver function		0.696		N.A	
Child-Pugh A	41		1		
Child-Pugh B	43		1.131 (0.623–2.054)		
TP73-AS1 expression		0.005		0.019	
Higher	42		2.427 (1.311–4.533)		2.249 (1.141–4.430)
Lower	42		1		1

### TP73-AS1 knockdown inhibited HCC cell proliferation and downregulated HMGB1 signal pathway

**Fig. 2:**
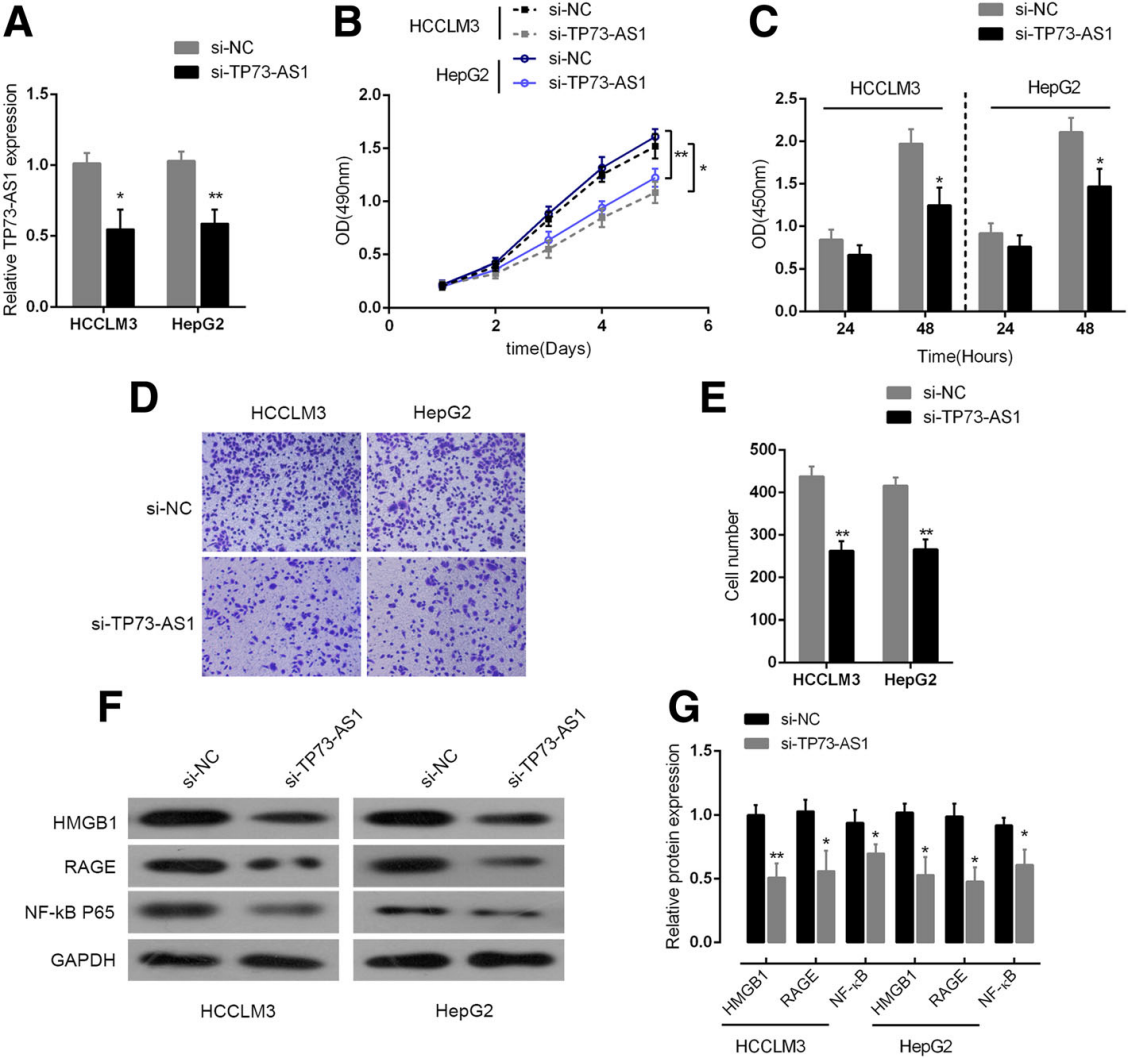
TP73-AS1 knockdown inhibited HCC cell proliferation and downregulated HMGB1 signal pathway **a** TP73-AS1 knockdown was achieved by si-TP73-AS1 and the inhibitory efficiency was verified by real-time PCR. **b** MTT assays was performed to determine the proliferation of HCC cell lines, HCCLM3 and HepG2, in response to knocking down of TP73-AS1, compared with si-NC group. **c** BrdU was performed to determine the proliferation of HCC cell lines, HCCLM3 and HepG2, in response to knocking down of TP73-AS1, compared with si-NC group. **d** and **e** Colony formation assay was performed to determine the colony formative capacity of HCC cell lines, HCCLM3 and HepG2, in response to knocking down of TP73-AS1, compared with si-NC group. **f** and **g** The protein levels of HMGB1, RAGE and NF-κB were determined in si-TP73-AS1-transfected HCCLM3 and HepG2 cells. The data are presented as mean ± SD of three independent experiments. *P < 0.05, **P < 0.01

Next we investigated the association of TP73-AS1 expression with HCC cell proliferation. TP73-AS1 knockdown in HepG2 and HCCLM3 was achieved by si-TP73-AS1 and the inhibitory efficiency was verified by real-time PCR (Fig. 2a). Then the cell proliferation was determined by MTT and BrdU assays. MTT and BrdU assays revealed that knocking down of TP73-AS1 significantly attenuated the proliferation of both HepG2 and HCCLM3 cell lines over time, compared with si-NC group (Fig. 2b and c). Colony formation assays revealed that knocking down of TP73-AS1 markedly reduced colony formative capacity of both HepG2 and HCCLM3 cell lines, and the cell numbers of HepG2 and HCCLM3 cell lines in si-TP73-AS1 group were significantly decreased compared with si-NC group (Fig. 2d and e).

High-mobility group box 1 protein (HMGB1) is an evolutionarily ancient and critical regulator of cell death and survival. According to previous studies, the ability of proliferation, migration and invasion of HCC cells was strengthened when the expression endogenous HMGB1 was enhanced using HMGB1 DNA [26]. HMGB1 activates RAGE signaling pathways and induces NF-кB activation to promote cellular proliferation, invasion, and metastasis, in HCC cell lines [27]. Here we monitored the protein levels of HMGB1, RAGE and NF-кB in response to TP73-AS1 knockdown in HepG2 and HCCLM3 cell lines by using Western blot assays. Results showed that in both HepG2 and HCCLM3 cell lines, the protein levels of HMGB1, RAGE and NF-кB were significantly downregulated after TP73-AS1 knockdown by si-TP73-AS1 transfection (Fig. 1f and g). Taken together, these data revealed that TP73-AS1 was specifically upregulated in HCC tissues and cell lines; TP73-AS1 knockdown inhibited HCC cell proliferation and downregulated HMGB1/RAGE signal pathway.

### MiR-200a could negatively regulate HMGB1 expression

**Fig. 3:**
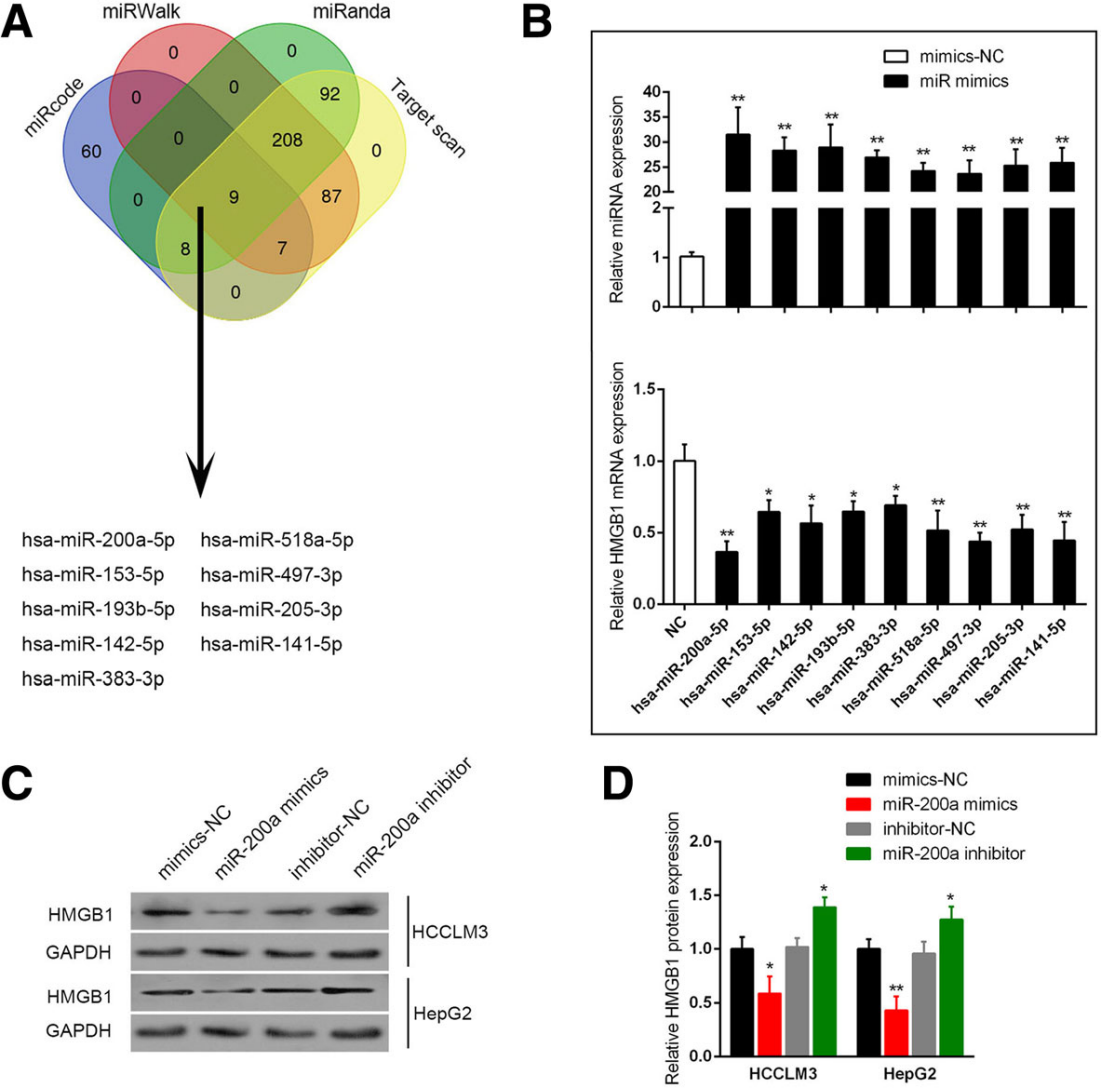
miR-200a could negatively regulate HMGB1 expression **a** Online tools including miRcode, miRWalk, miRanda and TargetScan were used to screen out the candidate upstream miRNAs of HMGB1. **b** The miRNA mimics of the indicated miRNAs were transfected into HepG2 cells, respectively. HMBG1 mRNA expression in response to ectopic miRNA expression of the indicated miRNAs was monitored by using real-time PCR. **c** and **d** The protein levels of HMGB1 in HCCLM3 and HepG2 cells in response to miR-200a overexpression or inhibition were determined by using Western blot assays. The data are presented as mean ± SD of three independent experiments. *P < 0.05, **P < 0.01

According to previous studies, several miRNAs were reported to inhibit HMGB1 expression to suppress tumor progress in many kinds of cancers [28, 29]. To further investigate the mechanism by which TP73-AS1 regulates HCC cell proliferation, we used online tools to screen out nine miRNAs which were potentially correlated with HMGB1 (miR-200a, miR-153, miR-193b, miR-142, miR-383, miR-518a, miR-497, miR-205 and miR-141) (Fig. 3a). HepG2 cells were transfected with the indicated miRNA mimics; the transfection efficiency was verified by using real-time PCR assays (Fig. 3b). Then HMGB1 mRNA expression in HepG2 cells was determined by using real-time PCR assays. Among the nine miRNAs, miR-200a inhibited HMGB1 mRNA the most significantly (Fig. 3b). The protein levels of HMGB1 in HepG2 and HCCLM3 cell lines in response to miR-200a overexpression and inhibition were then determined by using Western blot assays. Results showed that HMGB1 protein levels were reduced by miR-200a overexpression while increased by miR-200a inhibition (Fig. 3c and d). These data indicated that miR-200a could negatively regulate HMGB1 expression in HCC cell lines.

### TP73-AS1 competed with HMGB1 for miR-200a binding

**Fig. 4:**
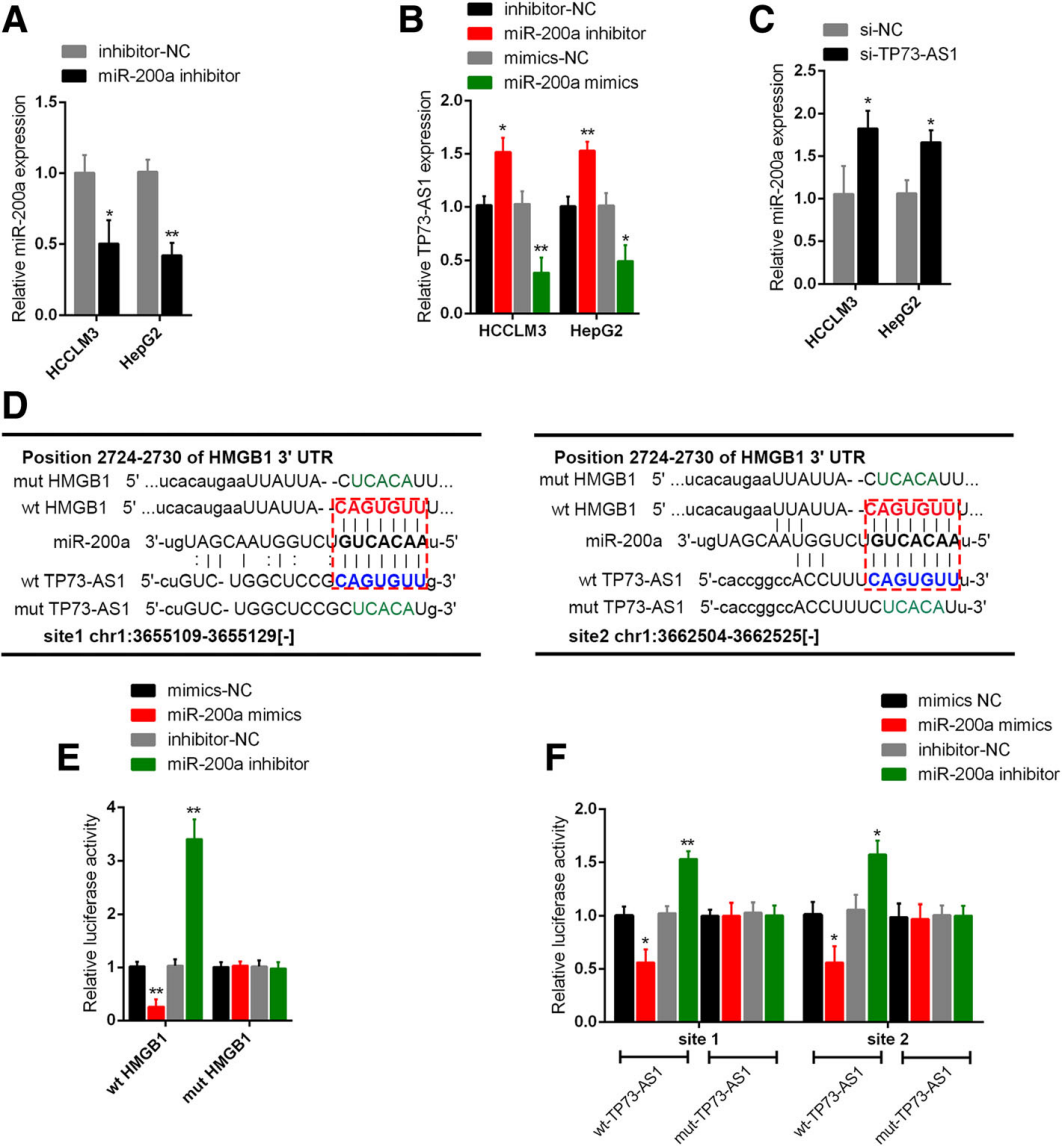
TP73-AS1 competed with HMGB1 for miR-200a binding **a** miR-200a inhibitor was transfected into HCCLM3 and HepG2 cells to achieve miR-200a inhibition. The inhibitory efficiency was verified by using real-time PCR. **b** TP73-AS1 expression in response to ectopic miR-200a expression and inhibition in HCCLM3 and HepG2 cells was determined by using real-time PCR. **c** miR-200a expression in response to TP73-AS1 knockdown in HCCLM3 and HepG2 cells was determined by using real-time PCR. **d** A wt-TP73-AS1 luciferase reporter vector (wt-TP73-AS1), as well as a mut-TP73-AS1 luciferase reporter vector (mut-TP73-AS1) by sequentially mutating the predicted two miR-200a binding sites in TP73-AS1 was constructed. A wt-HMGB1 3’UTR luciferase reporter vector (wt-HMGB1 3’UTR), as well as a mut-HMGB1 3’UTR luciferase reporter vector (mut-HMGB1 3’UTR) by sequentially mutating the predicted miR-200a binding sites in the 3’UTR of HMGB1 was constructed. **e** The wt-HMGB1 3’UTR/mut-HMGB1 3’UTR vectors and miR-200a inhibitor/miR-200a mimics were co-transfected into HepG2 cells. The luciferase activity of the wt- or mut-HMGB1 3’UTR luciferase reporter vector was determined by using dual luciferase assays. **f** The wt-TP73-AS1/mut-TP73-AS1 vectors and miR-200a inhibitor/miR-200a mimics were co-transfected into HepG2 cells. The luciferase activity of the wt- or mut-TP73-AS1 luciferase reporter vector was determined by using dual luciferase assays. The data are presented as mean ± SD of three independent experiments. *P < 0.05, **P < 0.01

We revealed that miR-200a negatively regulates HMGB1 expression; next we validated the correlation between TP73-AS1 and miR-200a. miR-200a inhibitor was transfected into HepG2 and HCCLM3 cell lines to achieve miR-200a inhibition (Fig. 4a). TP73-AS1 expression in response to miR-200a overexpression and inhibition in HepG2 and HCCLM3 cell lines was determined by using real-time PCR assays. Results showed that TP73-AS1 was upregulated by miR-200a inhibition while downregulated by miR-200a overexpression (Fig. 4b). After si-TP73-AS1-induced TP73-AS1 knockdown, miR-200a expression in HepG2 and HCCLM3 cell lines was increased (Fig. 4c). According to online tools, TP73-AS1 shared a same binding site in miR-200a with HMGB1. Next luciferase assays were arranged to figure out the correlations between miR-200a and HMGB1, between miR-200a and TP73-AS1. Luciferase reporter gene vectors (wt-HMGB1 3’UTR, mut-HMGB1 3’UTR containing a 7 bp mutation on miR-200a binding site in the 3’UTR of HMGB1, wt-TP73-AS1, and mut-TP73-AS1 containing a 7 bp mutation on miR-200a binding site1 or site2 in TP73-AS1) were constructed and co-transfected into HepG2 cells with miR-200a mimics or miR-200a inhibitor (Fig. 4d). The luciferase activity was then determined by using dual luciferase assays. Results showed that the luciferase activity of wt-HMGB1 3’UTR and wt-TP73-AS1 vectors was significantly reduced by miR-200a mimics while amplified by miR-200a inhibitor; these changes of luciferase activity were abolished after the predicted binding sites were mutated (Fig. 4e and f). These data indicated that both TP73-AS1 and HMGB1 could bind to miR-200a; TP73-AS1 competed with HMGB1 for miR-200a binding.

### TP73-AS1 regulated HMGB1/RAGE expression and NF-кB targets cytokines levels through miR-200a

**Fig. 5:**
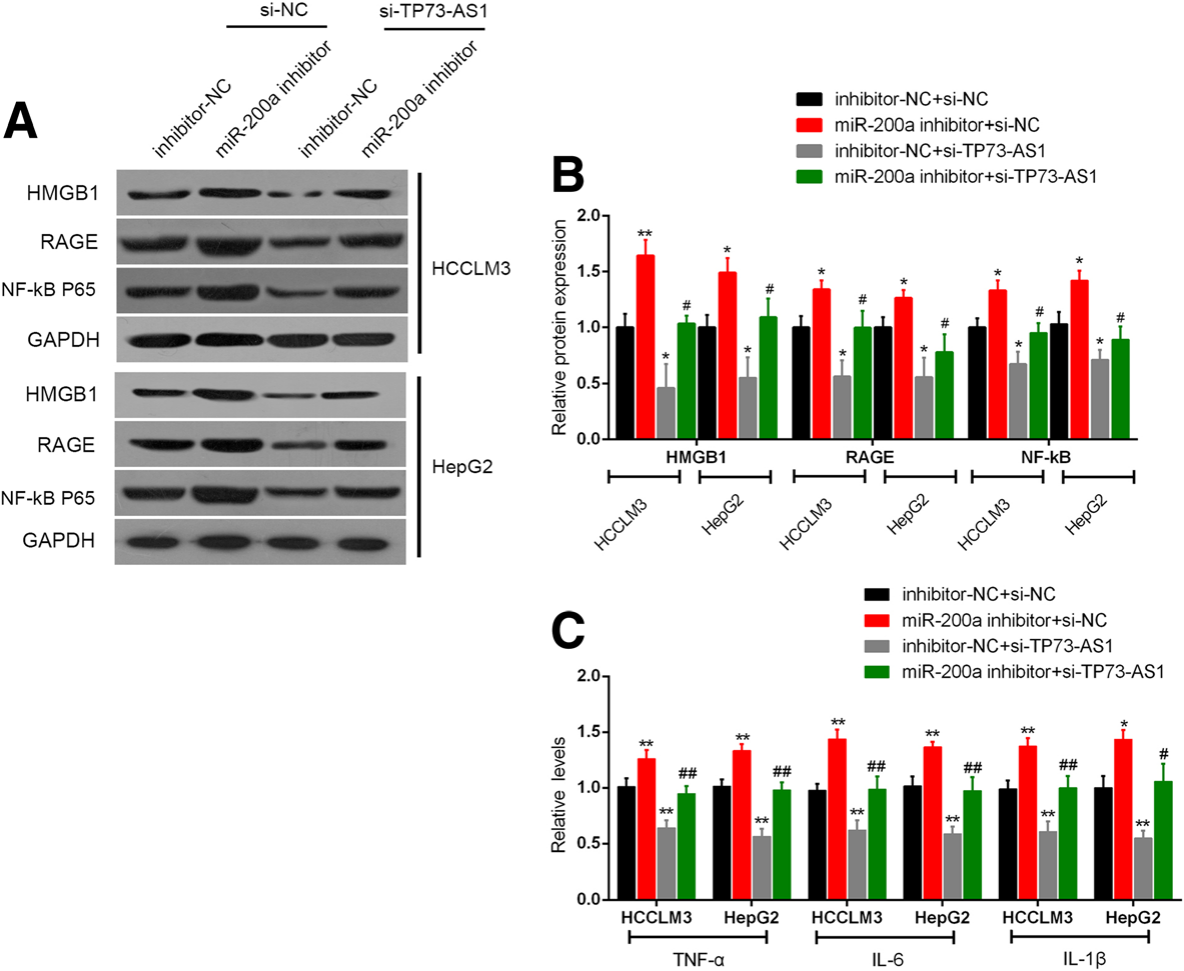
TP73-AS1 regulated HMGB1/RAGE expression and NF-κB targets cytokines levels through miR-200a **a** and **b** HepG2 and HCCLM3 cell lines were co-transfected with si-TP73-AS1 and miR-200a inhibitor. Then the protein levels of HMGB1, RAGE and NF-κB were determined by using Western blot assays. The gradation analysis of the Western blot films were performed using Image J software. **c** The levels of TNF-α, IL-6 and IL-1βin cultured media were measured by ELISA. The data are presented as mean ± SD of three independent experiments. *P < 0.05, **P < 0.01 vs. inhibitor-NC + si-NC group, # *P* < 0.05, ## *P* < 0.01 vs. inhibitor-NC + si-TP73-AS1 group

We demonstrated that TP73-AS1 knockdown inhibited the protein levels of HMGB1, RAGE and NF-кB; TP73-AS1 directly binds to miR-200a to inhibit miR-200a expression. Then we validated whether TP73-AS1 regulated HMGB1/RAGE expression through miR-200a. We co-transfected HepG2 and HCCLM3 cell lines with miR-200a inhibitor and si-TP73-AS1, and then monitored the protein levels of HMGB1, RAGE and NF-кB using Western blot assays and the gradation analysis. Results showed that in both HepG2 and HCCLM3 cell lines, the protein levels of HMGB1, RAGE and NF-кB were increased by miR-200a inhibition, reduced by si-TP73-AS1; the promotive effect of miR-200a inhibitor on the protein levels of HMGB1, RAGE and NF-кB could be partially abolished by si-TP73-AS1 (Fig. 5a and b). Moreover, as a transcription factor, NF-кB could promote the transcription of inflammatory cytokines [30]. Consistently with the NF-кB protein expression change trend, the ELISA assays demonstrated that levels of TNF-α, IL-6 andIL-1β were upregulated by miR-200a inhibition, reduced by si-TP73-AS1; the promotive effect of miR-200a inhibitor on the levels of TNF-α, IL-6 andIL-1β could be partially abolished by si-TP73-AS1 (Fig. 5c).

### Expression of miR-200a, HMGB1 and RAGE and the correlation of TP73-AS1 with miR-200a, HMGB1 and RAGE in HCC tissues

**Fig. 6:**
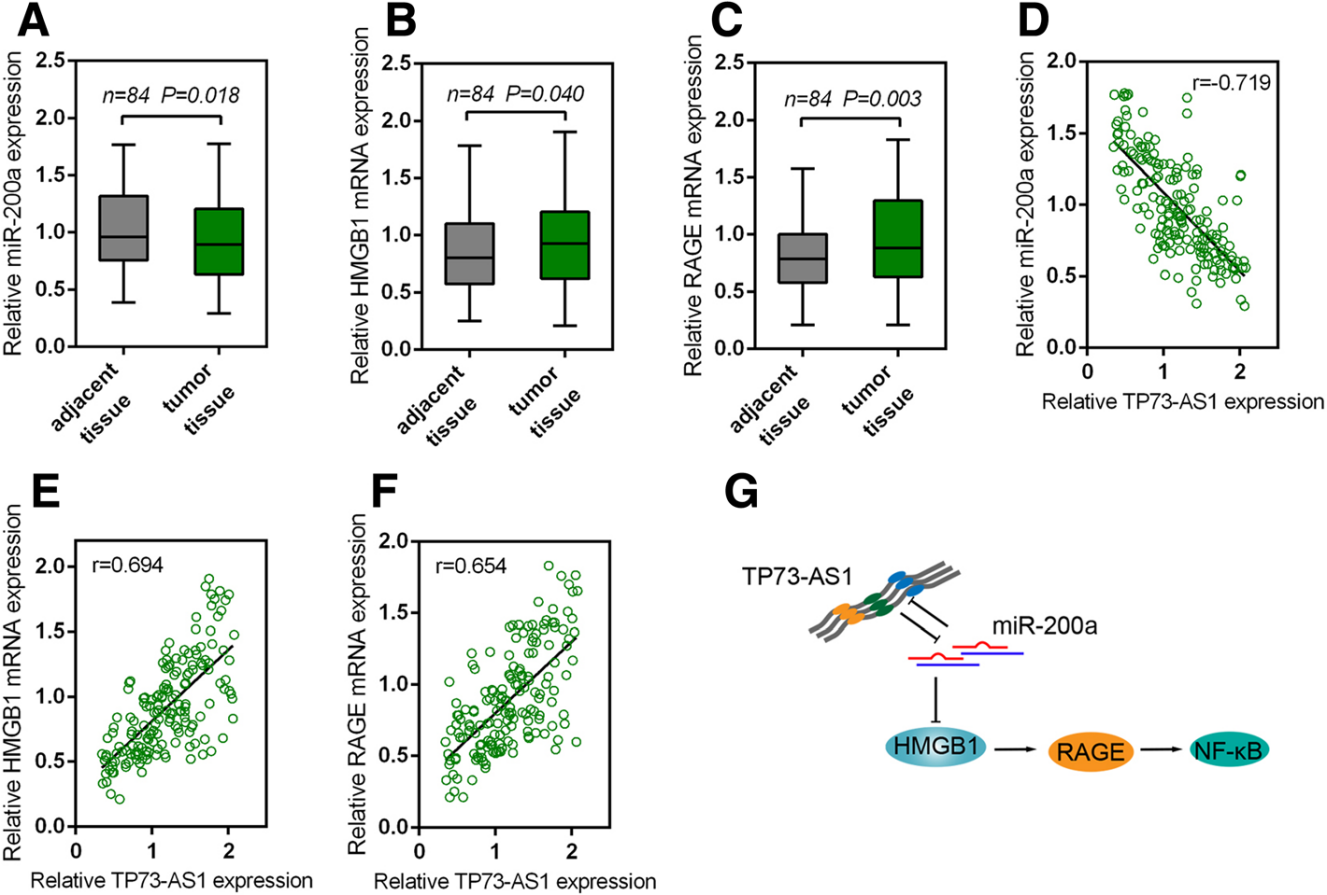
The correlation of TP73-AS1 with miR-200a, HMGB1 and RAGE in HCC tissues **a**, **b** and **c** The expression levels of miR-200a, HMGB1 mRNA and RAGE mRNA in 84 paired tumor tissues and adjacent normal tissues were determined by using real-time PCR. The data are presented as mean ± SD of three independent experiments. **d**, **e** and **f** The correlations between TP73-AS1 and miR-200a, HMGB1 and RAGE, respectively, were analyzed by using the Spearman’s rank correlation analysis. **g** Mechanism diagram of TP73-AS1 modulating HCC cell proliferation through miR-200a-dependent HMGB1/RAGE pathway regulation

We determined the expression levels of miR-200a, HMGB1 and RAGE in HCC tissues. Results showed that miR-200a expression was downregulated, while HMGB1 and RAGE expression was upregulated in HCC tissues (Fig. 6a, b and c). A negative correlation between TP73-AS1 and miR-200a expression levels, a positive correlation between TP73-AS1 and HMGB1 levels, and a positive correlation between TP73-AS1 and RAGE expression levels was observed (Fig. 6d, e and f). Taken together, we demonstrated that TP73-AS1 inhibited miR-200a to promote HCC cell proliferation through HMGB1/RAGE pathway.

## Discussion

The non-coding portion of the genome accounts for greater than 90% of the total mammalian genome. Studies have demonstrated that among these ncRNAs, ~18% of lncRNAs are associated with human tumors, compared with only 9% of human protein-coding genes [31], suggesting that lncRNAs could act as major contributors to carcinogenesis and cancer progression. The roles of dysregulated lncRNAs in the proliferation, invasion and migration of many kinds of cancers have garnered increased scientific interest in recent years [32–34].

LncRNA TP73-AS1 has been reported to be up-regulated in esophageal squamous cell carcinoma [18]. LncRNA TP73-AS1 knockdown inhibited BDH2 expression in EC9706 and KYSE30 cells, whereas BDH2 knockdown repressed esophageal cancer cell proliferation and induced apoptosis via the caspase-3 dependent apoptotic pathway [18]. In this study, we initially monitored TP73-AS1 expression in a large panel of 84 paired HCC tissues and adjacent normal tissues. A significant higher expression level of TP73-AS1 in HCC tissues was observed compared to the adjacent normal tissues. Consistently, the expression level of TP73-AS1 was obviously higher in HCC cell lines compared to that of the normal cell lines. According to the clinical information in 84 cases of patients with HCC, we found that TP73-AS1 expression was correlated with poor clinicopathological characteristics and was one of independent risk factors of overall survival. Moreover, TP73-AS1 might accelerate the HCC progress through promoting the proliferation of HCC cells. However, the mechanism by which TP73-AS1 affects the progression of HCC still remains to be validated.

HMGB1 and its receptor RAGE are pivotal factors in the development and progression of many types of tumor, including HCC [27]. Emerging evidences have demonstrated that chronic infection of HBV or HCV can induce the translocation of HMGB1 from nuclei to cytoplasm and extracellular release of HMGB1 [35, 36]. Serum HMGB1 levels were found significantly higher in HCC patients and overexpression of HMGB1 was associated with their clinico-pathological features and outcomes [37]. HMGB1 and RAGE can modulate the proliferation and cell cycle of HCC cells [38]. These findings show that HMGB1-RAGE axis may play an important role in the development of HCC, such as the proliferation of HCC cells. We revealed the promotive of TP73-AS1 on HCC proliferation, here we further validated whether TP73-AS1 could act on HMGB1/RAGE to affect HCC cell proliferation. In fact, we observed significantly reduced protein levels of HMGB1, RAGE and NF-κB in TP73-AS1-knocked down HCC cells.

MiRNAs expression dysfunctions are involved in almost all fields of cancer biology, including cell proliferation, invasion and migration. They can play the role of either tumor suppressors or oncogenes [39]. The mechanisms by which miRNAs exert their functions vary in different kinds of cancers, among which binding to the downstream target genes to inhibit their expression is a major one. Several miRNAs were reported to inhibit different downstream genes to delay the progress of HCC, including miR-636 (inhibiting Ras-PI3K/AKT pathway) [40], miR-16 (inhibiting COX-2) [41], miR-497 (inhibiting CHEK1) [42], and so on. Here, online tools were used to screen out the candidate upstream miRNAs for HMGB1. Among nine promising candidate miRNAs, we focused on miR-200a because of its essential regulator roles in breast cancer, skin tumor and ovarian tumorigenesis through targeting different downstream genes. MiR-200a suppresses cell growth and migration by targeting MACC1 in hepatocellular carcinoma [43]. In neuroblastoma cells, miR-200a inhibits tumor proliferation by targeting AP-2gamma [44]. Here, we found that miR-200a significantly down-regulated HMGB1 expression in HCC cells, which inspired us to hypothesize miR-200a might play some functional roles in HCC through regulation of HMGB1. More interestingly, a negative dual-regulation between miR-200a and TP73-AS1 was observed. As predicted by online tools, TP73-AS1 shared a similar binding site of miR-200a with HMGB1. By using luciferase assays, we confirmed miR-200a could directly bind to TP73-AS1 and the 3’UTR of HMGB1 on the predicted binding site(s). Given the consistency of the binding site(s), TP73-AS1 might compete with HMGB1 for miR-200a binding, so that to attenuate the inhibitory effect of miR-200a on HMGB1 expression.

To confirm that miR-200a regulated HMGB1 pathway in HCC cell lines, we monitored the protein levels of HMGB1, RAGE and NF-κB in response to the combined effect of miR-200a inhibition and TP73-AS1 knockdown. After miR-200a inhibited, the protein levels of HMGB1, RAGE and NF-κB were significantly upregulated. When TP73-AS1 was knocked out by si-TP73-AS1 in miR-200a inhibitor-transfected HCC cell lines, the promotive effect of miR-200a inhibitor on the protein levels of HMGB1, RAGE and NF-κB could be partially abolished. Moreover, as a transcription factor, NF-кB could promote the transcription of inflammatory cytokines [30]. Therefore, NF-κB targets cytokines TNF-α, IL-6 and IL-1β’s levels were also showed consistent change trends with NF-κB expression. These data indicated that in HCC cell lines, TP73-AS1 affected cell proliferation through miR-200a-dependent HMGB1/RAGE regulation. Moreover, in HCC tissues, miR-200a expression was downregulated, while HMGB1 and RAGE expression was upregulated. TP73-AS1 was inversely correlated with miR-200a, while positively correlated with HMGB1 and RAGE, respectively, which further confirmed that TP73-AS1 targeted miR-200a to inhibit its expression, subsequently upregulated HMGB1/RAGE expression to promote HCC cell proliferation.

## Conclusions

In summary, it was identified that TP73-AS1 could promote HCC cell proliferation through miR-200a-dependent HMGB1/RAGE regulation; TP73-AS1 might compete with HMGB1 for miR-200a binding to inhibit miR-200a expression. TP73-AS1 might be a key role in regulating HCC cells proliferation, and may provide a potential therapeutic target for HCC treatment.

## Additional file

Additional file 1: Table S1. Sequence of primers. (DOCX 17 kb)

## Abbreviations

BrdU: 5-Bromo-2-deoxyUridine
CXCL1: Chemokine (C-X-C motif) ligand 1
HCC: Hepatocellular carcinoma
lncRNA: Long non-coding RNA
MTT: 3-(4,5-dimethyl-2-thiazolyl)-2,5-diphenyl-2-H-tetrazolium bromide.
