# Supplementary material for: The long non-coding RNA TP73-AS1 modulates HCC cell proliferation through miR-200a-dependent HMGB1/RAGE regulation
Source: J Exp Clin Cancer Res. 2017 Apr 12;36:51. doi: 10.1186/s13046-017-0519-z (PMC5389141; doi:10.1186/s13046-017-0519-z)
Supplement: Additional file 1: Table S1. — Sequence of primers. (DOCX 17 kb) [file 13046_2017_519_MOESM1_ESM.docx]

Table S1 Sequence of primers

| Primers | Sequence |
| --- | --- |
| TP73-AS1 | F:GCTCCGTGAACCAACTCG  R:CCCTGCCAAGGGAACTCT |
| MiR-200a-5p | RT:GTCGTATCCAGTGCAGGGTCCGAGGTATTCGCACTGGATACGACTCCAGC  F:GGCGCATCTTACCGGACAGT |
| MiR-153-5p | RT:GTCGTATCCAGTGCAGGGTCCGAGGTATTCGCACTGGATACGACAGCTGC  F:GGCGTCATTTTTGTGATGTT |
| MiR-142-5p | RT:GTCGTATCCAGTGCAGGGTCCGAGGTATTCGCACTGGATACGACAGTAGT  F:GGCGCGCATAAAGTAGAAAGC |
| MiR-193b-5p | RT:GTCGTATCCAGTGCAGGGTCCGAGGTATTCGCACTGGATACGACTCATCT  F:GGCGCGGGGTTTTGAGGGCG |
| MiR-383-3p | RT:GTCGTATCCAGTGCAGGGTCCGAGGTATTCGCACTGGATACGACTCTGAC  F:GGCGCGACAGCACTGCCTG |
| MiR-518a-5p | RT:GTCGTATCCAGTGCAGGGTCCGAGGTATTCGCACTGGATACGACGAAAGG  F:GGCGCGCTGCAAAGGGAAGC |
| MiR-497-3p | RT:GTCGTATCCAGTGCAGGGTCCGAGGTATTCGCACTGGATACGACTCTAAC  F:GGCGCAAACCACACTGTGGT |
| MiR-205-3p | RT:GTCGTATCCAGTGCAGGGTCCGAGGTATTCGCACTGGATACGACGAACTT  F:GGCGCGGATTTCAGTGGAGTG |
| MiR-141-5p | RT:GTCGTATCCAGTGCAGGGTCCGAGGTATTCGCACTGGATACGACTCCAAC  F:GGCGCATCTTCCAGTACAGT |
| MiRNA universal reverse primer | GTGCAGGGTCCGAGGT |
| U6 | F:CTCGCTTCGGCAGCACA  R:AACGCTTCACGAATTTGCGT |
| HMGB1 | F:TATGGCAAAAGCGGACAAGG  R:CTTCGCAACATCACCAATGGA |
| RAGE | F:GTGTCCTTCCCAACGGCTC  R:ATTGCCTGGCACCGGAAAA |
| GAPDH | F:GGAGCGAGATCCCTCCAAAAT  R:GGCTGTTGTCATACTTCTCATGG |
